# Ultraviolet B Inhibits IL-17A/TNF-α-Stimulated Activation of Human Dermal Fibroblasts by Decreasing the Expression of IL-17RA and IL-17RC on Fibroblasts

**DOI:** 10.3389/fimmu.2017.00091

**Published:** 2017-02-03

**Authors:** Li Yin, YingYing Hu, JiaLi Xu, Jing Guo, Jie Tu, ZhiQiang Yin

**Affiliations:** ^1^Department of Dermatology, The First Affiliated Hospital of Nanjing Medical University, Nanjing, China; ^2^Department of Oncology, The First Affiliated Hospital of Nanjing Medical University, Nanjing, China

**Keywords:** ultraviolet, psoriasis, IL-17, TNF-α, fibroblast, TGF-β

## Abstract

**Background:**

Psoriasis is a chronic immune-mediated inflammatory disease, and a mixed Th1/Th17 cytokine environment plays a critical role in the pathogenesis of psoriasis. Dermal fibroblasts secrete certain cytokines such as IL-6, IL-8, and CXCL-1, contributing to the hyperproliferative state of the epidermis in psoriatic skin. Ultraviolet B (UVB) phototherapy is one of the most commonly used treatments in psoriasis but the influence of UVB on human dermal fibroblasts (HDFs) in psoriasis treatment is not completely understood.

**Objectives:**

We conducted this study to mimic a psoriatic microenvironment in order to investigate and illustrate the combined effects of UVB, IL-17A, and TNF-α on HDFs.

**Methods:**

The cultured HDFs were obtained from foreskin samples and divided into four groups, as follows: control; IL-17A/TNF-α; UVB; and IL-17A/TNF-α + UVB. Cultured HDFs were irradiated with 30 mJ/cm^2^ UVB followed by addition of IL-17A/TNF-α and incubated for 24 h. We used real-time quantitative PCR, Western blot, ELISA analysis, and flow cytometry to examine gene and protein expression of related pro-inflammatory cytokines and chemokines and receptors.

**Results:**

HDFs produced significant IL-6, IL-8, and CXCL-1 in response to IL-17A/TNF-α stimulation and UVB irradiation but UVB irradiation inhibited IL-17A/TNF-α-induced IL-6, IL-8, and CXCL-1 expression and downregulated the expression of IL-17RA and IL-17RC at both gene and protein levels. Additionally, UVB irradiation induced significant TGF-β1 protein secretion and expression of Smad3 mRNA and protein by HDFs. TGF-β1 significantly induced the expression of Smad3 mRNA and downregulated the IL-17RA and IL-17RC expression on HDFs.

**Conclusion:**

UVB irradiation inhibits IL-17A/TNF-α-induced IL-6, IL-8, and CXCL-1 production in HDFs by decreasing the expression of IL-17RA and IL-17RC on fibroblasts through TGF-β1/Smad3 signaling pathway, which reveals a new mechanism of the therapeutic action of UVB on psoriasis.

## Introduction

Psoriasis is a chronic immune-mediated inflammatory disease with complex cytokines network and diverse cellular participation, which mainly includes T lymphocytes, keratinocytes, neutrophils, antigen presenting cells, even fibroblasts, and so on. A mixed Th1/Th17 cytokine environment plays a critical role in the pathogenesis of psoriasis (1). TNF-α is a key cytokine in the development of psoriasis, and most psoriatic patients have a positive response to the anti-TNF biologics. TNF-α alone does not induce significant response from cultured keratinocytes; however, TNF-α combined with IL-17A forms intense synergies and amplifies the inflammatory reaction (2).

Fibroblasts are the principal cells of the dermis and emerging as an important player in inflammation (3). Dermal fibroblasts can secrete insulin-like growth factor-I that contribute to the epidermal hyperplasia of psoriasis by promoting keratinocyte proliferation (4). Dermal fibroblasts can also secrete certain cytokines such as IL-6, IL-8, and CXCL-1, which contribute to the hyperproliferative state of the epidermis in psoriatic skin, and these cytokines production in fibroblasts would increase *via* interaction with neutrophils or normal/malignant epithelial cells, or incubation in the presence of TNF-α or white adipocytes-secreted leptin (5–8). As one of the most important cytokines in psoriasis, IL-17A increases IL-8 and MCP-1 production in a dose-dependent manner in human dermal fibroblasts (HDFs) both at protein and mRNA levels, and there exists synergistic activity between IL-17A and TNF-α (9).

Ultraviolet B (UVB) irradiation mainly affects the epidermis and the superficial dermis. UVB phototherapy is one of the oldest and most commonly used treatments in psoriasis, including heliotherapy, broadband UVB, narrowband UVB, and excimer laser (10). It is generally recognized that UVB has an immunosuppressive effect on the skin. UVB induces lymphocyte apoptosis and alleviates psoriasis *via* the upregulation of regulatory T cells (11). Additionally, UVB treatment causes a marked reduction in the expression of skin-homing molecules by circulating T cells (12).

However, the influence of UVB on HDFs potently involved in the pathogenesis of psoriasis is not completely understood in psoriasis phototherapy. Hence, we conducted this study to mimic a psoriatic microenvironment in order to investigate and illustrate the combined effects of UVB, IL-17A, and TNF-α on HDFs.

## Materials and Methods

### Ethics Statement

This study was carried out in accordance with the recommendations of institutional guidelines and Local Ethics Committee of the First Affiliated Hospital of Nanjing Medical University with written informed consent from all subjects.

### Cell Culture for HDFs

The foreskins of healthy adult (age range 18–60 years) were the source of HDFs. HDFs were isolated and cultured as previously described (13). Circumcised prepuce samples were obtained in accordance with the ethical committee approval process of Jiangsu Province Hospital. All subjects gave written informed consent in accordance with the Declaration of Helsinki. HDFs used for experiments were in the second or third passage.

### Treatment of HDFs

Recombinant human IL-17A (100 ng/ml), TNF-α (10 ng/ml), and TGF-β1 (2 ng/ml) were obtained from PEPROTECH (Rocky Hill, NJ, USA), and P144 (2 µg/ml) was purchased from MedChem Express (Monmouth Junction, NJ, USA). The source of UVB was a BLE-1T158 UV lamp (Spectronics Corp., Westbury, NY, USA). The UVB dosage (30 mJ/cm^2^) was quantified using a Waldmann UV meter (model number 585,100; Herbert Waldmann GmbH & Co., KG, Villingen-Schwenningen, Germany). The HDFs were seeded in 6-well culture plates and incubated for 24 h. Experiments were repeated independently at least three times.

### Real-time Quantitative PCR

After 24 h of incubation, the supernatant was collected, and the HDFs attached to the culture plates were washed three times using PBS and then dissolved in TRIzol^®^ reagent (Invitrogen; Life Technologies Corp., Carlsbad, CA, USA), and total RNA was isolated. First-strand cDNA was synthesized from 2 µg of total RNA: RNA was incubated at 42°C for 1 h with M-MuLV Reverse Transcriptase (Thermo Fisher Scientific Inc., Waltham, MA, USA) following oligo(dT) priming, and then the enzyme was denatured at 70°C for 10 min. PCR amplification was performed in a total volume of 20 µl containing 1 µl template cDNA, and transcripts quantified using StepOnePlus™ Real-Time PCR System (Applied Biosystems, USA). All values were normalized to the expression of the housekeeping gene *GAPDH*. Primer sequences are shown in Table 1.

**Table 1:** **Primers for the target genes in real-time quantitative PCR**.

Target genes	Primers
*GAPDH* (115 bp)	Sense: 5-CATCTTCTTTTGCGTCGCCA-3
	Antisense: 5-TTAAAAGCAGCCCTGGTGACC-3
*IL-6* (120 bp)	Sense: 5-GACAGCCACTCACCTCTTCA-3
	Antisense: 5-CCTCTTTGCTGCTTTCACAC-3
*IL-8* (149 bp)	Sense: 5-GCAGAGGGTTGTGGAGAAGT-3
	Antisense: 5-CCCTACAACAGACCCACACA-3
*CXCL-1* (126 bp)	Sense: 5-AATCCAACTGACCAGAAGGG-3
	Antisense: 5-CATTAGGCACAATCCAGGTG-3
*TNFR-1* (98 bp)	Sense: 5-TACCGGCATTATTGGAGTGA-3
	Antisense: 5-GTGTTCTGTTTCTCCTGGCA-3
*TNFR-2* (109 bp)	Sense: 5-ACACCGTGTGTGACTCCTGT-3
	Antisense: 5-TGAGTTTCCACCTGGTCAGA-3
*IL-17RA* (128 bp)	Sense: 5-GCTGCCTTTGTCCTCCTAAC-3
	Antisense: 5- GACTGACTGTGCTGATGGCT -3
*IL-17RC* (84 bp)	Sense: 5-CAGAAGGAGACCGACTGTGA-3
	Antisense: 5-CTCATCTTCAGGCTCTTCCC-3
*TGF-β1* (128 bp)	Sense: 5-AACCGGCCTTTCCTGCTTCT-3
	Antisense: 5-CGCACGCAGCAGTTCTTCTC-3
*Smad3* (85 bp)	Sense: 5-ATGCAGCAGTGGAGCTGACA-3
	Antisense: 5-AGGCACTCTGCGAAGACCTC-3

### Western Blot for IL-6, IL-8, CXCL-1, and Smad3 Expression of HDFs

After 24 h, the HDFs were washed and then lysed in RIPA buffer containing protease inhibitor. Centrifugal separation was conducted at 4°C, at 14,000 rpm for 15 min. The upper layer of the solution was tested for protein using the Bradford method. SDS-PAGE was performed. The primary antibody was added as below: IL-6 (ab9324), IL-8 (ab106350), β-actin (ab8226), Smad3 (ab84177) antibodies (Abcam, Cambridge, UK), and CXCL-1 (Novus Biologicals, Littleton, CO, USA), following the manufacturer’s instructions. Differences in protein expression were examined using Gel-Pro Analyzer 32 (Media Cybernetics, Rockville, MD, USA).

### ELISA Analysis of IL-6, IL-8, CXCL-1, and TGF-β1 Secretion in Culture Supernatant

Measurement of these secretory protein was performed using IL-6, IL-8, TGF-β1 ELISA kits (ExCell Bio, Shanghai, China), and CXCL-1 ELISA kit (CUSABIO, Wuhan, Hubei, China). This assay uses the quantitative sandwich immunoassay technique.

### Flow Cytometry for Detection of the Surface Expression of IL-17RA and IL-17RC on HDFs

Detection of IL-17RA and IL-17RC expression were performed using human IL-17RA-FITC and IL-17RC-FITC antibodies (Miltenyi Biotec, Bergisch Gladbach, Germany). HDFs were fixed with 70% alcohol, washed twice with PBS, digested with RNase, and stained with propidium iodide. A flow cytometer (FAC-Scan, BD, NJ, USA) was used to gather data and images.

### Tissue Culture and Immunohistochemistry

Three individuals with chronic plaque psoriasis were enrolled (age range 18–60 years). Inclusion criteria included no systemic antipsoriatic treatments for 1 month before biopsy. Biopsies were taken from psoriatic plaques. All subjects gave written informed consent according to the Declaration of Helsinki.

The subdermal tissue was removed by scraping with forceps, and each tissue cut into two pieces of 0.5–1.0 cm, and then cultured dermal side down in 1 ml RPMI with 10% fetal bovine serum, 55 mM β-mercaptoethanol, 2 mM glutamine, 100 µg/ml streptomycin, and 100 U/ml penicillin per well of 12-well culture plate for 24 h (14). The epidermis was kept above the medium surface. Then, 60 mJ/cm^2^ of UVB was irradiated once to the epidermal side of the tissues before culture.

After 24 h, the tissues were collected and formalin fixed and paraffin embedded for immunohistochemical staining. Slides were prepared using a Ventana autoimmunostainer (Loche, USA) and available IL-6, IL-8 antibodies (Proteintech, Wuhan, Hubei, China) and CXCL-1 antibody (Bioworld, Nanjing, Jiangsu, China).

The numbers of typical staining positive fibroblasts were counted for five successive fields in high magnification (HM, ×400). The number of cells was calculated and expressed as the numbers of Positive fibroblasts/HM.

### Statistical Analysis

GraphPad Prism for Windows (GraphPad Software, San Diego, CA, USA) was used for data analysis. All data are presented as mean ± SD. Data were tested for normality and statistical significance calculated using a Student’s *t*-test, Mann–Whitney *U* test, or Friedman’s test, as appropriate. Significance was defined as *P* < 0.05.

## Results

### IL-17A/TNF-α Stimulation Have Additive or Synergistic Effect on Pro-inflammatory Cytokines and Chemokines Expression by HDFs

**Figure 1:**
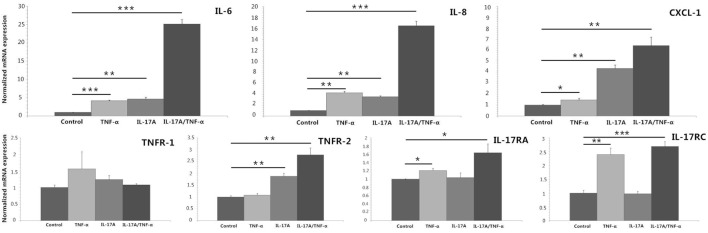
**Real-time quantitative PCR analyses of HDFs stimulated with IL-17A (100 ng/ml) and TNF-α (10 ng/ml) alone or together for 24 h. The results demonstrate that both IL-17A and TNF-α induce significant increases in IL-6, IL-8, and CXCL-1 mRNA expression and IL-17A/TNF-α stimulation have additive or synergistic effect on IL-6, IL-8, and CXCL-1 mRNA expression**. Additionally, IL-17A induces the mRNA expression of TNFR-2 and TNF-α induces IL-17RA and IL-17RC expression on HDFs. The data (fold change) are from one representative experiment performed in triplicate, repeated three times with similar results. HDFs, human dermal fibroblasts. Statistical significance indicated: *P < 0.05, **P < 0.01, ***P < 0.001.

We stimulated HDFs with IL-17A and TNF-α alone or together, which showed both IL-17A and TNF-α induced significant increases in IL-6, IL-8, and CXCL-1 mRNA expression (*P* < 0.05; Figure 1). In addition, synergistic (IL-6 and IL-8) and additive (CXCL-1) effect of IL-17A/TNF-α stimulation on HDFs were seen. Our study also showed IL-17A induced the mRNA expression of TNFR-2 and TNF-α induced IL-17RA and IL-17RC expression on HDFs (*P* < 0.05; Figure 1).

### HDFs Produce Significant IL-6, IL-8, and CXCL-1 in Response to IL-17A/TNF-α Stimulation and UVB Irradiation

**Figure 2:**
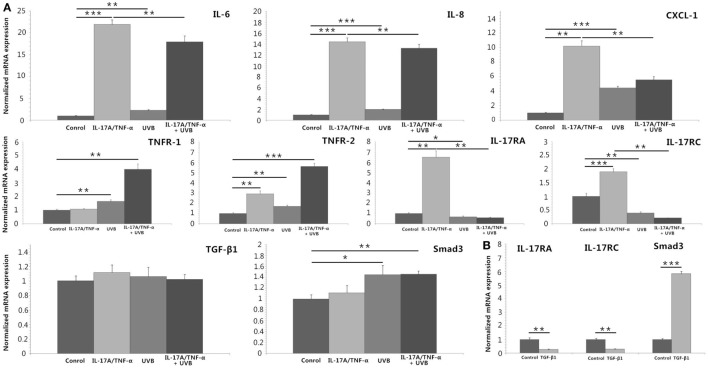
**Real-time quantitative PCR analyses of HDFs treated with IL-17A (100 ng/ml)/TNF-α (10 ng/ml), UVB (30 mJ/cm^2^), and TGF-β1 (2 ng/ml) for 24 h**. **(A)** IL-17A/TNF-α-stimulated HDFs increase expression of IL-6, IL-8, and CXCL-1 mRNA, which are also seen in 30 mJ/cm^2^ UVB-irradiated HDFs. But UVB irradiation inhibits IL-17A/TNF-α-induced IL-6, IL-8, and CXCL-1 mRNA expression of HDFs. IL-17A/TNF-α stimulation induce the expression of TNFR-2, IL-17RA, and IL-17RC mRNA. UVB irradiation upregulates the expression of TNFR-1 and TNFR-2 mRNA but downregulates IL-17RA and IL-17RC expression, and inhibits IL-17A/TNF-α-induced IL-17RA and IL-17RC mRNA expression. IL-17A/TNF-α and UVB treatment do not induce significant expression of TGF-β1 mRNA 24 h after culture, but Smad3 mRNA is upregulated in both UVB irradiation and IL-17A/TNF-α + UVB groups. **(B)** TGF-β1 significantly induces the Smad3 mRNA expression and downregulates the IL-17RA and IL-17RC expression in HDFs. The data (fold change) are from one representative experiment performed in triplicate, repeated three times with similar results. UVB, ultraviolet B; HDFs, human dermal fibroblasts. Statistical significance indicated: *P < 0.05, **P < 0.01, ***P < 0.001.

**Figure 3:**
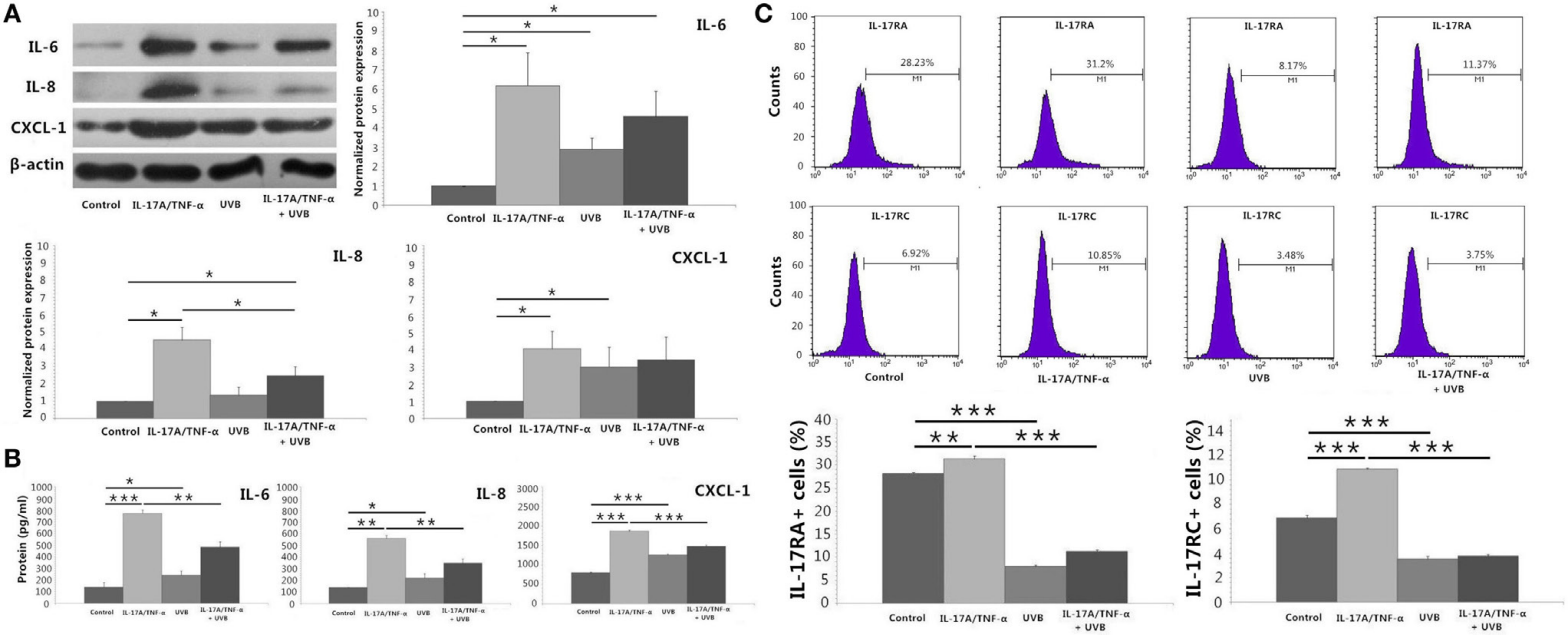
**Protein detection by using Western blot, ELISA analysis, and flow cytometry**. **(A)** Western blot of HDFs lysates confirms mRNA expression of IL-6, IL-8, and CXCL-1 at the protein level, although shows no significant increase in IL-8 protein of UVB group compared with Control group and no significant decrease in IL-6 and CXCL-1 protein of IL-17A/TNF-α + UVB group compared with IL-17A/TNF-α group. **(B)** ELISA analysis of the supernatant confirms mRNA expression of IL-6, IL-8, and CXCL-1 at the protein level. **(C)** The expression of IL-17RA and IL-17RC mRNA is confirmed by flow cytometry for detection of the surface expression of IL-17RA and IL-17RC on HDFs. The data (fold change) of Western blot are expressed as the mean ± SD of three independent experiments. Other data are from one representative experiment performed in triplicate, repeated three times with similar results. UVB, ultraviolet B; HDFs, human dermal fibroblasts. Statistical significance indicated: *P < 0.05, **P < 0.01, ***P < 0.001.

We stimulated HDFs with IL-17A/TNF-α and observed statistically significant increases in IL-6, IL-8, and CXCL-1 mRNA. Treatment of HDFs with UVB irradiation also led to marked mRNA expression of IL-6, IL-8, and CXCL-1 (*P* < 0.01; Figure 2A). The increase in IL-6, IL-8, and CXCL-1 was further confirmed at the protein level (Figures 3A,B). Although Western blot of cell lysates did not show significant increase in IL-8 protein in UVB group, ELISA analysis of supernatant confirmed the IL-8 protein secretion. HDFs irradiated with UVB followed by IL-17A/TNF-α stimulation expressed significantly less IL-6, IL-8, and CXCL-1 mRNA compared with single IL-17A/TNF-α treatment (*P* < 0.01; Figure 2A), which was also confirmed at the protein level (Figures 3A,B). Similarly, Western blot did not show significant decrease in IL-6 and CXCL-1 protein in IL-17A/TNF-α + UVB group compared with IL-17A/TNF-α group, but ELISA analysis confirmed the significant difference of protein secretion.

### The Inhibitory Effect of UVB on IL-17A/TNF-α-Stimulated Activation of HDFs Is through Decreasing the Expression of IL-17RA and IL-17RC on Fibroblasts

We investigated the expression of TNFR-1, TNFR-2, IL-17RA, and IL-17RC mRNA, which showed IL-17A/TNF-α stimulation increased the mRNA expression of TNFR-2, IL-17RA, and IL-17RC by HDFs and UVB irradiation increased the expression of TNFR-1 and TNFR-2 (*P* < 0.01; Figure 2A) but decreased IL-17RA and IL-17RC expression (*P* < 0.05; Figure 2A). IL-17A/TNF-α combined with UVB treatment had synergistic effect on TNFR-1 and TNFR-2 mRNA expression by HDFs, however, induced significant decrease in IL-17RA and IL-17RC mRNA in comparison with single IL-17A/TNF-α stimulation (*P* < 0.01; Figure 2A). The decrease in IL-17RA and IL-17RC mRNA was further confirmed by flow cytometry for detection of the surface expression of IL-17RA and IL-17RC on HDFs (Figure 3C).

### UVB (30 mJ/cm^2^) Irradiation Induces Significant TGF-β1 Protein Secretion and Expression of Smad3 mRNA and Protein by HDFs

**Figure 4:**
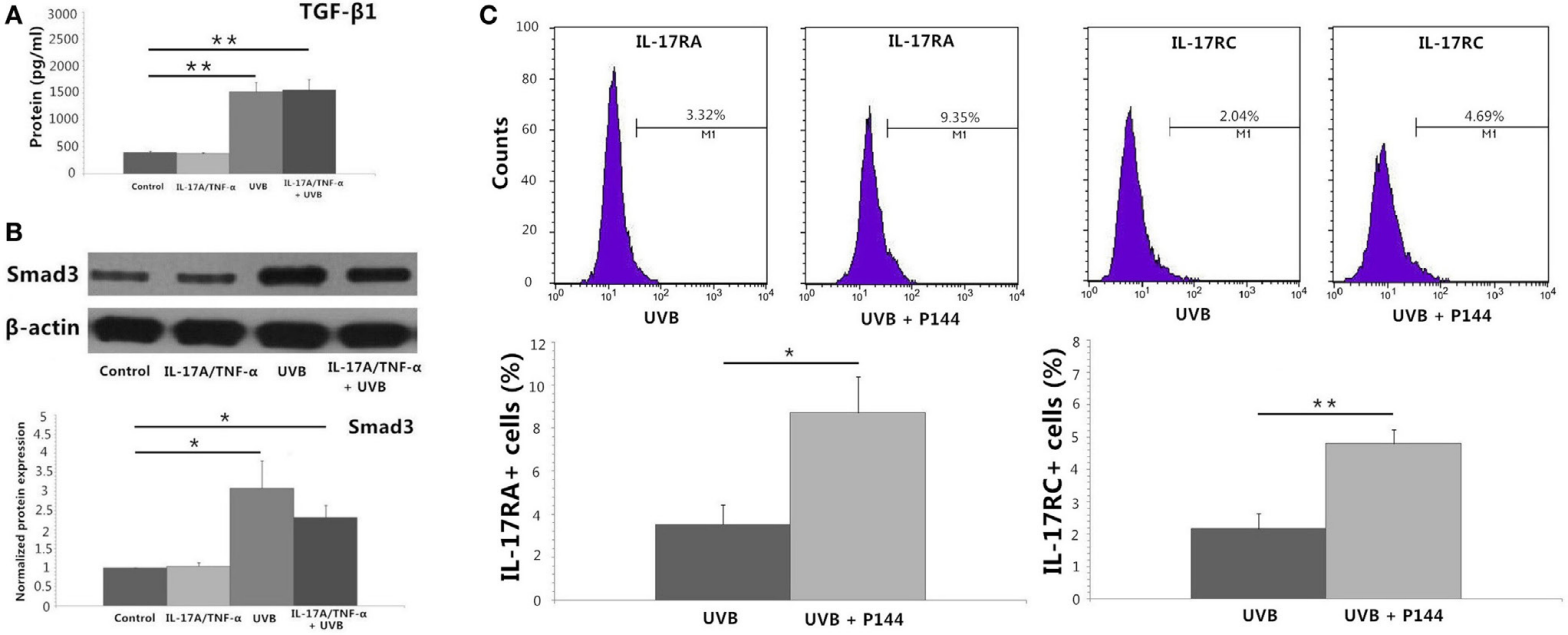
**Protein detection by using ELISA analysis, Western blot, and flow cytometry**. **(A)** ELISA analysis shows significant TGF-β1 protein secretion in supernatant in both UVB and IL-17A/TNF-α + UVB groups. **(B)** Western blot shows the protein expression of Smad3 is significantly upregulated in both UVB and IL-17A/TNF-α + UVB groups. **(C)** Flow cytometry shows P144 (2 µg/ml) is able to block the inhibitory effect of UVB on the expression of IL-17RA and IL-17RC on HDFs. The data (fold change) of Western blot are expressed as the mean ± SD of three independent experiments. Other data are from one representative experiment performed in triplicate, repeated three times with similar results. UVB, ultraviolet B; HDFs, human dermal fibroblasts. Statistical significance indicated: *P < 0.05, **P < 0.01.

IL-17A/TNF-α and UVB (30 mJ/cm^2^) did not induce significant expression of TGF-β1 mRNA 24 h after treatment (Figure 2A), but ELISA analysis showed significant TGF-β1 protein secretion in supernatant in both UVB and IL-17A/TNF-α + UVB groups compared with Control group (*P* < 0.01; Figure 4A). The mRNA and protein expression of Smad3 were simultaneously upregulated in both UVB and IL-17A/TNF-α + UVB groups (*P* < 0.05; Figures 2A and 4B).

We used recombinant human TGF-β1 (2 ng/ml) to stimulate HDFs for 24 h, which showed TGF-β1 significantly induced the Smad3 mRNA expression and downregulated the IL-17RA and IL-17RC expression on HDFs (*P* < 0.01; Figure 2B). We also observed P144 (anti-TGF-β1) blocked the inhibitory effect of UVB on the expression of IL-17RA and IL-17RC on fibroblasts (*P* < 0.05; Figure 4C).

### UVB (60 mJ/cm^2^) Irradiation Decreases the Expression of IL-6, IL-8, and CXCL-1 in Dermal Fibroblasts of Psoriatic Plaques

**Figure 5:**
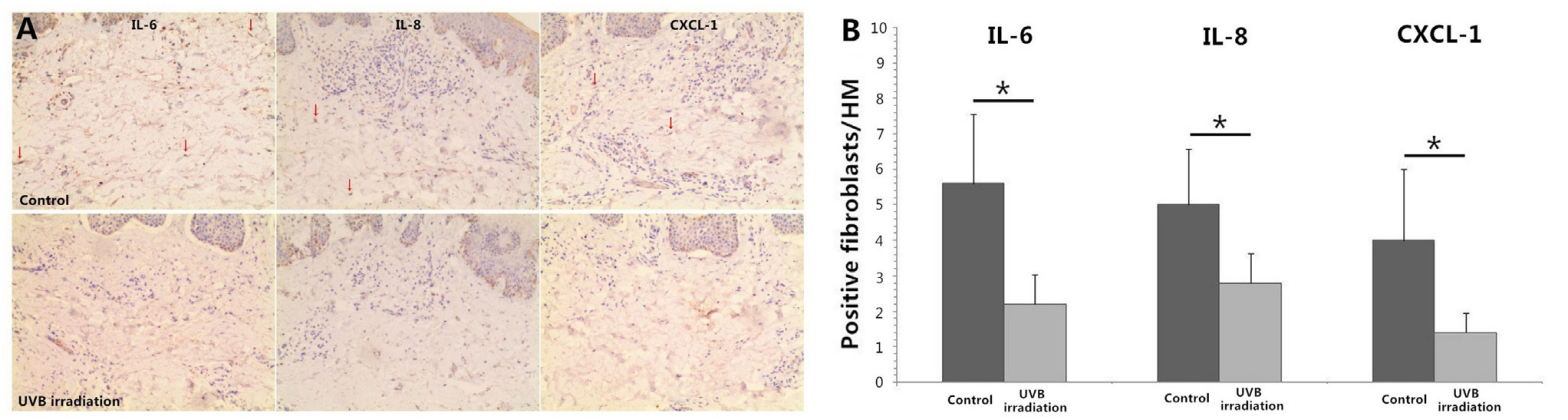
**Immunohistochemical staining of IL-6, IL-8, and CXCL-1 in psoriatic plaque skin**. **(A)** More staining positive dermal fibroblasts are seen in dermis of Control group (unirradiated psoriatic plaque skin) but less staining positive fibroblasts in UVB (60 mJ/cm^2^) irradiation group (*n* = 3), original magnification ×200. *Red arrows*, some typical staining positive fibroblasts. **(B)** The numbers of typical staining positive fibroblasts are counted for five successive fields in HM (×400), which confirms the significant difference between Control group and UVB irradiation group. The data are from one representative psoriatic skin and other two psoriatic skin have similar results. UVB, ultraviolet B; HM, high magnification. Statistical significance indicated: *P < 0.05.

Immunohistochemical staining of IL-6, IL-8, and CXCL-1 showed multiple cells in dermis were staining positive including vascular endothelial cell, lymphocytes, and fibroblasts. More staining positive dermal fibroblasts were seen in dermis of unirradiated psoriatic plaque lesion. In UVB irradiation group, there were less staining positive fibroblasts (*P* < 0.05; Figure 5).

## Discussion

As the key cytokines in the pathogenesis of psoriasis, IL-17A can synergize with TNF-α to induce the production of MCP-1, IL-8, and MMP-1 by dermal fibroblasts (9). Our study proved IL-17A (100 ng/ml) and TNF-α (10 ng/ml) stimulation had synergistic or additive effect on the expression of IL-6, IL-8, and CXCL-1 mRNA and protein by HDFs. IL-6, IL-8, and CXCL-1 play active roles in the development of psoriasis (6). On the other hand, HDFs subjected to UVB irradiation induce significant TNF-α, IL-6, and IL-8 production (15, 16). All together, we speculated prior to the onset of our experiment that there might be additive or synergistic effect of IL-17A/TNF-α stimulation combined with UVB irradiation on pro-inflammatory cytokines and chemokines expression by HDFs. Interestingly, our HDFs study showed that there was no synergy between IL-17A/TNF-α and UVB, and unexpectedly UVB significantly inhibited IL-17A/TNF-α-induced IL-6, IL-8, and CXCL-1 expression by HDFs, which were confirmed at both gene and protein levels.

IL-17A signals through a receptor complex composed of IL-17RA and IL-17RC, which are expressed on keratinocytes, endothelial cells, fibroblasts, and so on (17). TNFR-1 (p55) and TNFR-2 (p75) on dermal fibroblasts are involved in TNF-α-mediated DNA synthesis and IL-6 and IL-8 release (18). TNF-α has the ability to increase the expression of IL-17R by keratinocytes and IL-17A to induce TNFR expression, and short hairpin RNA inhibition of IL-17R expression abrogates synergistic gene induction by TNF-α and IL-17A (1, 19). Human primary lung fibroblasts stimulated with TNF-α expressed increased mRNA levels of IL-17BR (IL-17RA–IL-17RB complex) (20). UVB induces the expression of TNFR-1 and TNFR-2 on murine fibroblasts in a dose-dependent manner (21). Our study observed TNF-α and IL-17A mutually induced the mRNA expression of another cytokine’s receptor on HDFs and IL-17A/TNF-α-stimulated HDFs had significant elevated mRNA expression of TNFR-2, IL-17RA, and IL-17RC. We also found that UVB irradiation increased the expression of TNFR-1 and TNFR-2 on HDFs, which was consistent with the findings on murine fibroblasts (21). Additionally, IL-17A/TNF-α and UVB had synergistic effect on mRNA expression of TNFR-1 and TNFR-2 of HDFs. The effect of UVB irradiation on IL-17RA and IL-17RC of HDFs was interesting, where the mRNA and protein expression of IL-17RA and IL-17RC markedly decreased after low-dose UVB irradiation. Based on the above findings, we presumed that UVB suppressed IL-17RA and IL-17RC expression in HDFs, blunting the strong synergy between IL-17A and TNF-α, which contributed to decreased IL-6, IL-8, and CXCL-1 expression by HDFs.

TGF-β1 downregulated the expression of IL-17BR mRNA on human primary lung fibroblasts (20). Nakashima et al. reported ectopic TGF-β1 stimulation significantly downregulated the IL-17RA expression in normal fibroblasts and the effect of TGF-β1 on IL-17RA was Smad3 dependent (22). Our study confirmed TGF-β1-stimulated HDFs significantly upregulated Smad3 mRNA and downregulated IL-17RA and also IL-17RC, indicating that IL-17RA and IL-RC expression may be regulated by TGF-β1/Smad3 signaling pathway. The effect of UVB irradiation on TGF-β1 expression of HDFs depends on the dose of irradiation and observing time after treatment. HDFs were given 10 exposures to UVB at 250 mJ/cm^2^ (twice a day for 5 days) and TGF-β1 expression was significantly increased after the last stress (23). Then, 144 mJ/cm^2^ of UVB-irradiated HDFs secreted significantly less TGF-β1 protein 72 h after treatment compared with unirradiated cells (24). Choi et al. reported after irradiation with 25 mJ/cm^2^ UVB on HDFs, TGF-β1 mRNA significantly decreased at 24 and 48 h (25). Cao et al. investigated the expression of TGF-β1 mRNA in HDFs at various time points after 10 mJ/cm^2^ UVB irradiation and found TGF-β1 mRNA was increased 4 and 8 h after UVB irradiation and gradually decreased and reached the control level at 24 h, additionally, ELISA analysis showed TGF-β1 protein in the supernatant was increased 12 h after 10 mJ/cm^2^ UVB irradiation (26). Our study found there was no significant difference in the expression of TGF-β1 mRNA between 30 mJ/cm^2^ UVB-irradiated and unirradiated HDFs 24 h after treatment, but ELISA analysis of TGF-β1 showed significant increase in protein secretion of UVB irradiation group, indicating the increase in TGF-β1 mRNA expression after 30 mJ/cm^2^ UVB irradiation happens within 24 h. P144, a TGF-β1 antagonist peptide (27), was proved to be able to block the inhibition of UVB irradiation on the expression of IL-17RA and IL-17RC on HDFs. As above, the effect of UVB irradiation on HDFs seems to be changeable in different conditions, and low-dose UVB usually used for phototherapy has transient action on HDFs to induce TGF-β1 production and then inhibits the expression of IL-17RA and IL-17RC through TGF-β1/Smad3 signaling pathway.

IL-17A and TNF-α play key roles in the complex inflammatory network of psoriasis, promoting immune activation, and the maintenance of psoriasis lesions. Therapeutic options for psoriasis mainly include topical and systemic medications, biologic agents, and phototherapy. Phototherapy remains one of the most effective and safest model of treatment for psoriasis, especially UVB (10, 28). Our results confirm that UVB irradiation inhibits IL-17A/TNF-α-induced IL-6, IL-8, and CXCL-1 production in HDFs by decreasing the expression of IL-17RA and IL-17RC on fibroblasts through TGF-β1/Smad3 pathway, which reveals a new mechanism of the therapeutic action of UVB on psoriasis. Further investigation focusing on the effect of UVB irradiation on dermal fibroblasts isolated from psoriatic patients would be informative and necessary. The modes of action by which TNF-α and IL-17A mutually induce the expression of another cytokine’s receptor and UVB induces TNFR-1 and TNFR-2 expression on HDFs remains to be elucidated.

## Author Contributions

LY and YH responsible for cells culture and qRT-PCR and Western blot. JX responsible for flow cytometry and data analysis. JG and JT responsible for ELISA analysis. ZY responsible for the quality of overall manuscript. LY, YH, and JX contributed equally to this work.

## Conflict of Interest Statement

The authors declare that the research was conducted in the absence of any commercial or financial relationships that could be construed as a potential conflict of interest.

## References

[1] Baliwag J Barnes DH Johnston A. Cytokines in psoriasis. Cytokine (2015) 73(2):342–50. 10.1016/j.cyto.2014.12.014 25585875 PMC4437803

[2] Chiricozzi A Guttman-Yassky E Suarez-Farinas M Nograles KE Tian S Cardinale I Integrative responses to IL-17 and TNF-alpha in human keratinocytes account for key inflammatory pathogenic circuits in psoriasis. J Invest Dermatol (2011) 131:677–87. 10.1038/jid.2010.340 21085185

[3] Flavell SJ Hou TZ Lax S Filer AD Salmon M Buckley CD. Fibroblasts as novel therapeutic targets in chronic inflammation. Br J Pharmacol (2008) 153(Suppl 1):S241–6. 10.1038/sj.bjp.0707487 17965753 PMC2268075

[4] Miura H Sano S Higashiyama M Yoshikawa K Itami S. Involvement of insulin-like growth factor-I in psoriasis as a paracrine growth factor: dermal fibroblasts play a regulatory role in developing psoriatic lesions. Arch Dermatol Res (2000) 292(12):590–7. 10.1007/s004030000188 11214819

[5] Glowacka E Lewkowicz P Rotsztejn H Zalewska A. IL-8, IL-12 and IL-10 cytokines generation by neutrophils, fibroblasts and neutrophils–fibroblasts interaction in psoriasis. Adv Med Sci (2010) 55(2):254–60. 10.2478/v10039-010-0037-0 20934961

[6] Kolář M Szabo P Dvořánková B Lacina L Gabius HJ Strnad H Upregulation of IL-6, IL-8 and CXCL-1 production in dermal fibroblasts by normal/malignant epithelial cells in vitro: immunohistochemical and transcriptomic analyses. Biol Cell (2012) 104(12):738–51. 10.1111/boc.201200018 23043537

[7] Zalewska A Głowacka E Wyczółkowska J Tchórzewski H Narbutt J Sysa-Jedrzejowska A. Interleukin 6 and 8 levels in plasma and fibroblast cultures in psoriasis. Mediators Inflamm (2006) 2006(1):81767. 10.1155/MI/2006/81767 16864908 PMC1570391

[8] Ommen P Stjernholm T Kragstrup T Raaby L Johansen C Stenderup K The role of leptin in psoriasis comprises a proinflammatory response by the dermal fibroblast. Br J Dermatol (2016) 174(1):187–90. 10.1111/bjd.13969 26119283

[9] Brembilla NC Montanari E Truchetet ME Raschi E Meroni P Chizzolini C. Th17 cells favor inflammatory responses while inhibiting type I collagen deposition by dermal fibroblasts: differential effects in healthy and systemic sclerosis fibroblasts. Arthritis Res Ther (2013) 15(5):R151. 10.1186/ar4334 24289089 PMC3979123

[10] Matos TR Ling TC Sheth V. Ultraviolet B radiation therapy for psoriasis: pursuing the optimal regime. Clin Dermatol (2016) 34(5):587–93. 10.1016/j.clindermatol.2016.05.008 27638437

[11] Zhang D Chen Y Chen L Yang R Wang L Liu W Ultraviolet irradiation promotes FOXP3 transcription via p53 in psoriasis. Exp Dermatol (2016) 25(7):513–8. 10.1111/exd.12942 26781862

[12] Sigmundsdottir H Gudjonsson JE Valdimarsson H. The effects of ultraviolet B treatment on the expression of adhesion molecules by circulating T lymphocytes in psoriasis. Br J Dermatol (2003) 148(5):996–1000. 10.1046/j.1365-2133.2003.05318.x 12786832

[13] Zhang JA Zhou BR Xu Y Chen X Liu J Gozali M MiR-23a-depressed autophagy is a participant in PUVA- and UVB-induced premature senescence. Oncotarget (2016) 7:37420–35. 10.18632/oncotarget.9357 27191270 PMC5122322

[14] Yin Z Xu J Zhang Z Luo D. Effects of topical pimecrolimus 1% on high-dose ultraviolet B-irradiated epidermal Langerhans cells. Int Immunopharmacol (2012) 14(4):635–40. 10.1016/j.intimp.2012.10.002 23079131

[15] Karthikeyan R Kanimozhi G Prasad NR Agilan B Ganesan M Mohana S 7-Hydroxycoumarin prevents UVB-induced activation of NF-κB and subsequent overexpression of matrix metalloproteinases and inflammatory markers in human dermal fibroblast cells. J Photochem Photobiol B (2016) 161:170–6. 10.1016/j.jphotobiol.2016.04.027 27240190

[16] Kim JK Mun S Kim MS Kim MB Sa BK Hwang JK. 5,7-Dimethoxyflavone, an activator of PPARα/γ, inhibits UVB-induced MMP expression in human skin fibroblast cells. Exp Dermatol (2012) 21(3):211–6. 10.1111/j.1600-0625.2011.01435.x 22379967

[17] Ramirez-Carrozzi V Sambandam A Luis E Lin Z Jeet S Lesch J IL-17C regulates the innate immune function of epithelial cells in an autocrine manner. Nat Immunol (2011) 12(12):1159–66. 10.1038/ni.2156 21993848

[18] Butler DM Feldmann M Di Padova F Brennan FM. p55 and p75 tumor necrosis factor receptors are expressed and mediate common functions in synovial fibroblasts and other fibroblasts. Eur Cytokine Netw (1994) 5(5):441–8. 7880974

[19] Johnston A Guzman AM Swindell WR Wang F Kang S Gudjonsson JE. Early tissue responses in psoriasis to the antitumour necrosis factor-α biologic etanercept suggest reduced interleukin-17 receptor expression and signalling. Br J Dermatol (2014) 171(1):97–107. 10.1111/bjd.12937 24601997 PMC4115021

[20] Létuvé S Lajoie-Kadoch S Audusseau S Rothenberg ME Fiset PO Ludwig MS IL-17E upregulates the expression of proinflammatory cytokines in lung fibroblasts. J Allergy Clin Immunol (2006) 117(3):590–6. 10.1016/j.jaci.2005.10.025 16522458

[21] Kimura H Minakami H Shoji A. Ultraviolet B irradiation modulates susceptibility to tumour necrosis factor-alpha-induced apoptosis via induction of death receptors in murine fibroblasts. Cell Biol Int (2001) 25(12):1221–8. 10.1006/cbir.2001.0805 11748915

[22] Nakashima T Jinnin M Yamane K Honda N Kajihara I Makino T Impaired IL-17 signaling pathway contributes to the increased collagen expression in scleroderma fibroblasts. J Immunol (2012) 188(8):3573–83. 10.4049/jimmunol.1100591 22403442

[23] Debacq-Chainiaux F Borlon C Pascal T Royer V Eliaers F Ninane N Repeated exposure of human skin fibroblasts to UVB at subcytotoxic level triggers premature senescence through the TGF-beta1 signaling pathway. J Cell Sci (2005) 118(Pt 4):743–58. 10.1242/jcs.01651 15671065

[24] Sun Z Hwang E Park SY Zhang M Gao W Lin P *Angelica archangelia* prevented collagen degradation by blocking production of matrix metalloproteinases in UVB-exposed dermal fibroblasts. Photochem Photobiol (2016) 92(4):604–10. 10.1111/php.12595 27128690

[25] Choi CP Kim YI Lee JW Lee MH. The effect of narrowband ultraviolet B on the expression of matrix metalloproteinase-1, transforming growth factor-beta1 and type I collagen in human skin fibroblasts. Clin Exp Dermatol (2007) 32(2):180–5. 10.1111/j.1365-2230.2006.02309.x 17137474

[26] Cao Y Ohwatari N Matsumoto T Kosaka M Ohtsuru A Yamashita S. TGF-beta1 mediates 70-kDa heat shock protein induction due to ultraviolet irradiation in human skin fibroblasts. Pflugers Arch (1999) 438(3):239–44. 10.1007/s004240050905 10398851

[27] Gallo-Oller G Vollmann-Zwerenz A Meléndez B Rey JA Hau P Dotor J P144, a transforming growth factor beta inhibitor peptide, generates antitumoral effects and modifies SMAD7 and SKI levels in human glioblastoma cell lines. Cancer Lett (2016) 381(1):67–75. 10.1016/j.canlet.2016.07.029 27473823

[28] Mehta D Lim HW. Ultraviolet B phototherapy for psoriasis: review of practical guidelines. Am J Clin Dermatol (2016) 17(2):125–33. 10.1007/s40257-016-0176-6 26872953

